# Chronic Total Occlusions: Current Approaches, Evidence and Outcomes

**DOI:** 10.3390/jcm14134695

**Published:** 2025-07-02

**Authors:** Remi Arnold, Richard Gervasoni, Florence Leclercq

**Affiliations:** Department of Cardiology, Arnaud de Villeneuve Hospital, 34295 Montpellier, France; arnold.remi34@orange.fr (R.A.); r-gervasoni@chu-montpellier.fr (R.G.)

**Keywords:** coronary chronic total occlusion, percutaneous coronary intervention, major coronary events, procedure success

## Abstract

Chronic total occlusions (CTOs), defined as complete coronary artery blockages persisting for over three months, are frequently encountered in up to 25% of coronary angiograms. Although percutaneous coronary intervention (PCI) for CTO remains technically challenging, advancements in guidewires, microcatheters, re-entry devices, and intravascular imaging, along with the expertise of specialized operators, have significantly improved procedural success rates, now exceeding 90% in expert centers. While recent evidence, such as the SYNTAX II study, emphasizes the importance of complete revascularization, over half of CTO cases continue to be managed conservatively with optimal medical therapy (OMT), partly due to the limited high-quality randomized evidence supporting revascularization. Observational studies have demonstrated that successful CTO-PCI is associated with improved angina relief, quality of life, left ventricular function, and possibly long-term survival. Extended observational follow-up, such as the Korean and Canadian registries, suggests long-term reductions in cardiac and all-cause mortality with CTO revascularization. However, randomized controlled trials (RCTs) have primarily shown symptomatic benefit, with no consistent reduction in major adverse cardiac events (MACE) or mortality, likely due to limited sample sizes, short follow-up, and treatment crossovers. Various strategies, including the hybrid algorithm, guide CTO interventions by balancing antegrade and retrograde techniques based on lesion complexity. Imaging modalities such as coronary CT angiography and intravascular ultrasound play a pivotal role in planning and optimizing these procedures. Future innovations, such as real-time fusion imaging of CCTA with coronary angiography, may enhance lesion visualization and guidewire navigation. While current guidelines recommend CTO-PCI in selected symptomatic patients with demonstrable ischemia or viable myocardium, the decision should be individualized, incorporating anatomical feasibility, comorbidities, patient preferences, and input from a multidisciplinary Heart Team. Looking ahead, adequately powered RCTs with extended follow-up are essential to determine the long-term clinical impact of CTO-PCI on hard outcomes such as mortality and myocardial infarction.

## 1. Introduction

Chronic total occlusions (CTOs) are defined as a thrombolysis in myocardial infarction (TIMI) grade 0 flow within the occluded segment of a coronary artery with a >2.5 mm diameter, with an occlusion duration > 3 months [1].

These lesions are frequently encountered in interventional cardiology, observed in 15 to 25% of coronary angiograms [2,3]. Percutaneous coronary interventions (PCIs) for CTOs are still considered challenging procedures. However, the development of specialized techniques and equipment, including intracoronary imaging, along with the expertise of dedicated operators, has led to an increase in the recanalization success rate of up to 90% [4]. Whereas the results of the recent large SYNTAX II study [5] support the need for complete revascularization, in more than half of the patients, CTOs are managed with medical treatment [6]. Therefore, strong evidence is required to support the use of percutaneous revascularization in selected patients. The aim of this article is to provide a review of the current literature on this topic.

## 2. Clinical Presentation

The presence of collateral circulation at coronary angiograms is characteristic of CTOs. While these collaterals supply the myocardium at rest, preserving its viability, they are often insufficient during physical stress [7], leading to symptoms such as angina or dyspnea during exertion. In most cases, left ventricular ejection (LVEF) is preserved, and the electrocardiogram (ECG) appears normal [3]. Other symptomatic clinical presentations may be acute coronary syndromes, such as STEMI in the case of an acute occlusion in the artery supplying collaterals to the chronically occluded artery, as well as NSTEMI, including type 2 myocardial infarction, where patients present with troponin elevation and/or ECG changes due to functional myocardial ischemia [8]. A CTO can also be identified during a coronary angiogram when the culprit lesion is a non-CTO lesion.

While most patients present with symptoms, only a few remain asymptomatic (around 20%) [2]. In these cases, a diagnostic coronary angiogram is performed following positive non-invasive tests (stress-induced echocardiography or stress-induced SPECT) or after finding abnormal wall motion on transthoracic echocardiography (TTE). These patients typically present with common cardiovascular risk factors, including male sex, age over 60, diabetes, and history of myocardial infarction or PCI [9].

## 3. CTO Techniques and Strategy Algorithms

CTO interventions should be performed by experienced operators during a programmed and dedicated procedure. A well-trained team is essential to ensure a safe environment, where the operator has access to specialized equipment in order to improve success rates, while also being prepared to manage any potential complications.

**Figure 1:**
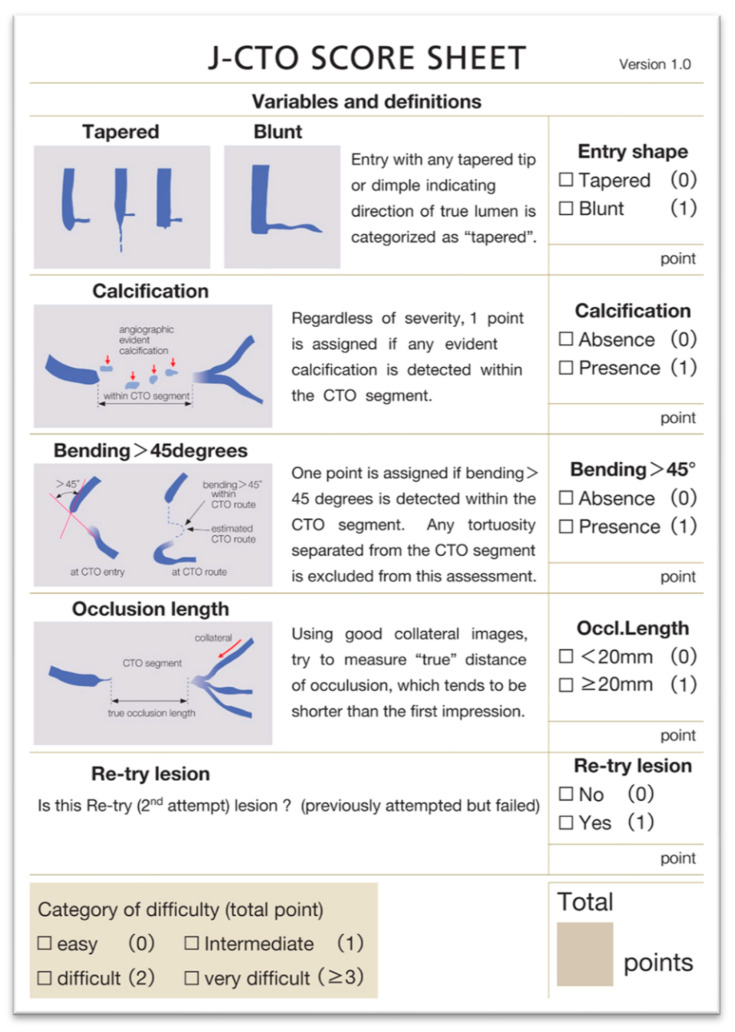
J-CTO score from Morino Y et al. [10].

Preparing and planning the revascularization strategy is a key point in CTO practice. Several scores have been developed to predict the difficulty of CTO procedures, with the Japanese CTO score (J-CTO score) being the most widely used. This score is based on 5 items and ranges from 0 to 5 and helps to predict the likelihood of successful anterograde wiring crossing [10] (Figure 1).

A meticulous analysis of the diagnostic coronary angiogram is necessary to properly visualize the collaterals and understand the CTO anatomy. In this regard, coronary computed tomography angiography (CCTA) can be very helpful. Pre-procedural CCTA can enhance the success rate of recanalization and minimize the risk of coronary perforations, especially in complex lesions with a J-CTO score ≥ 2 [11].

CTO experts [12] recommend dual coronary injection. Indeed, it allows visualization of the collaterals and minimizes perforation risk by helping determine guidewire position in the occluded segment.

A variety of specialized techniques have been developed to optimize success in CTO percutaneous coronary interventions. Generally, these techniques are categorized into anterograde and retrograde approaches.

**Figure 2:**
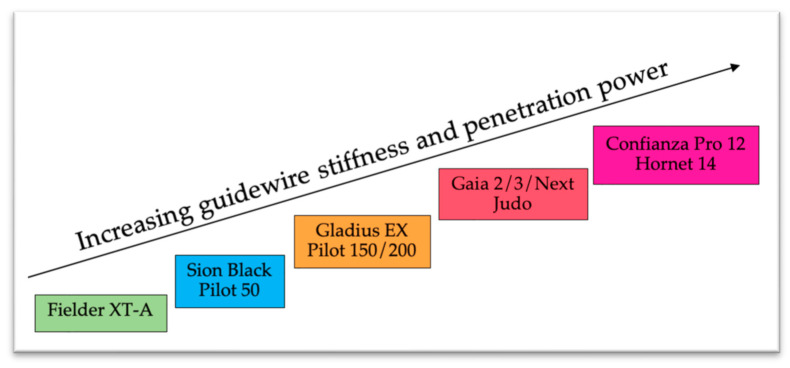
Proposed algorithm for the AWE technique.

The most commonly used is antegrade wire escalation (AWE) [13], which involves progressively stiffer guidewires to cross the occlusion through the true lumen (Figure 2). This technique is adequate in short lesions (occlusion length < 20 mm), with a non-ambiguous proximal cap and a good-quality distal vessel [14].

For complex CTO lesions, particularly those that are calcified, tortuous, or involve long-segment occlusions, true lumen wiring remains a significant challenge. In such scenarios, the Antegrade Dissection and Re-entry (ADR) technique can be beneficial. This method involves controlled navigation through the subintimal space using a polymer-jacketed guidewire in a “knuckled” configuration to facilitate advancement. Re-entry into the true lumen is then achieved using a specialized device, such as the Stingray balloon, which is inflated within the subintimal space to guide precise puncture back into the true lumen [15]. In addition to device-based approaches, several wire-based techniques are frequently used in clinical practice. In the scratch-and-go technique, a high tip-load guidewire is employed to directly access the extraplaque space, followed by microcatheter advancement into the subintimal plane. The stiff wire is then exchanged for a polymer-jacketed guidewire, which is used in a knuckled configuration to facilitate subintimal tracking. Alternatively, the balloon-assisted subintimal entry (BASE) technique involves dilation of a slightly oversized balloon just proximal to the occlusion, intentionally disrupting the intimal layer [15]. This disruption enables entry into the extraplaque space using a guidewire supported by a microcatheter, which is then followed by knuckle wire advancement. These wire-based methods broaden the applicability of ADR, particularly in scenarios where device-based re-entry is not feasible.

When antegrade approaches are unsuccessful or deemed unsuitable, the retrograde technique becomes a valuable alternative, particularly effective in long or ambiguous occlusions.

**Figure 3:**
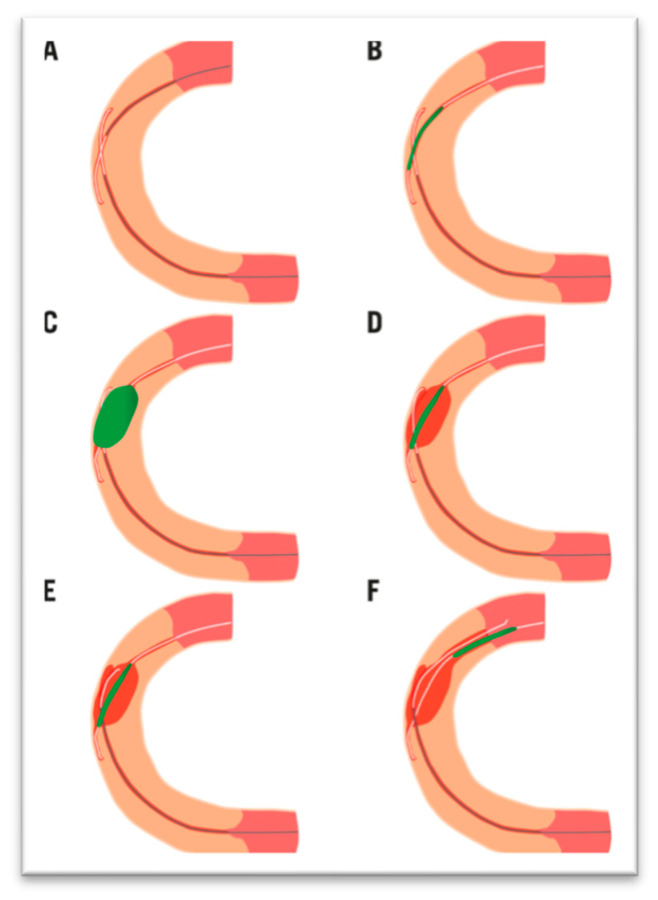
Illustration of the reverse CART technique from Patel VG et al. [17]. (**A**) Proximal RCA CTO (in pale orange) with overlapping antegrade and retrograde guidewires in a longitudinal orientation. (**B**) Balloon (in green) positioned over the antegrade guidewire at the site of overlap. (**C**) Balloon inflation at the overlap zone. (**D**) Creation of a connection between the subintimal spaces (in red) of both guidewires following balloon dilatation. (**E**) Advancement of the retrograde guidewire through the established connection. (**F**) Retrograde guidewire within the proximal true lumen.

Collateral assessment is a critical first step in planning a retrograde CTO approach, with the choice of an appropriate “interventional” collateral pathway, preferably a septal over epicardial route due to lower risk of severe complications, being guided by factors like collateral size, tortuosity, and operator experience [16]. Collateral wiring must be performed with extreme care to prevent damage to delicate vessels, using techniques such as septal surfing or selective injection, though surfing is not recommended for epicardial collaterals. Once the wire crosses, a microcatheter is advanced into the distal vessel. Various techniques can be employed to connect the antegrade and retrograde lumens. In the Retrograde Dissection Re-entry (RDR) technique, knuckled guidewires are advanced from both directions until they overlap within the occluded segment. When the retrograde wire is unable to reach the proximal true lumen, the reverse CART technique is utilized. This involves advancing both antegrade and retrograde wires simultaneously, followed by balloon dilation to enable the retrograde wire to enter the proximal vessel (Figure 3).

Modern CTO practice is often guided by algorithmic approaches, such as the Hybrid Algorithm [18], which emphasizes early dual coronary injection, rapid technique escalation, and real-time decision-making to minimize procedural time and complications. Initial strategy selection is guided by the assessment of three anatomical criteria: proximal cap ambiguity, poor distal target, and the presence of suitable interventional collaterals. When anatomy is not suitable, a retrograde approach is. Conversely, in the absence of these features, an antegrade strategy is favored: AWE for occlusions <20 mm in length and ADR for longer occlusions.

The Minimalistic Hybrid Algorithm proposed by Zivelonghi [19] aims to provide a less invasive alternative to traditional CTO PCI strategies by minimizing the use of dual access, large-bore catheters, and transfemoral approaches while preserving the full range of techniques from the conventional hybrid algorithm. It begins with thorough angiographic assessment to classify the CTO as either Simple or Complex, based on proximal cap clarity, presence of microchannels, collateral flow, and established CTO scoring systems (e.g., J-CTO score). High-quality contralateral injection is essential and should be obtained if not available at baseline. Simple CTOs typically show a clear proximal cap and favorable anatomy (J-CTO ≤ 1), allowing for a primary antegrade wire escalation (AWE) strategy using soft guidewires and microcatheters, usually via single 6F transradial access. If unsuccessful, the feasibility of retrograde intervention is reassessed. If suitable collaterals exist, a second radial access is added for a retrograde approach, enabling reverse CART or retrograde wire escalation (RWE). If retrograde access fails or is less favorable, escalation to advanced antegrade techniques (e.g., ADR with CrossBoss-Stingray) is performed, requiring upsizing to 7–8F access. Complex CTOs (J-CTO > 1) often have proximal cap ambiguity and other challenging features. If interventional collaterals are available, initial retrograde access via 6F radial is attempted. Upon successful distal cap crossing, an antegrade approach is added to complete revascularization with RWE or reverse CART. If retrograde options are not viable, ADR with larger-bore access is considered.

These techniques, when employed by experienced operators with access to dedicated tools and imaging modalities like IVUS, have significantly improved procedural success rates and safety in CTO interventions [20]. Intravascular ultrasound (IVUS) can be helpful after lesion crossing for stent sizing, detecting the presence of calcifications, and ensuring optimal stent deployment. Also, it can be useful for crossing ambiguous proximal caps and ostial occlusions and, in some cases, to confirm wire position in ADR or retrograde techniques.

The Asia-Pacific CTO algorithm provides a globally relevant alternative to the hybrid strategy, placing greater emphasis on comprehensive lesion characteristics rather than solely occlusion length. It prioritizes the anterograde approach, incorporating techniques such as IVUS-guided wiring in challenging cases and the use of parallel wiring or the Stingray system following unsuccessful anterograde wire escalation (AWE) [21].

A key challenge in CTO practice is the ability to adapt and change the crossing strategy during the intervention. This flexibility is crucial to maximize the chances of success while minimizing complications, radiation exposure, and contrast volume. Experts highlight the 5 reasons to stop a CTO attempt [12]: (1) occurrence of complications, (2) high radiation exposure (>5 Gy), (3) excessive contrast volume (>3.7× the estimated creatinine clearance), (4) exhaustion of crossing options, and (5) patient or physician fatigue.

## 4. CTO Procedures Challenges and Complications

CTO interventions were initially met with caution due to the perceived risk of complications. However, in the hands of experienced operators, the complication rate is now relatively low. In a meta-analysis by Patel et al. [22] involving 18,061 patients who underwent CTO interventions, the most common complications were contrast-induced nephropathy (3.8%), coronary perforation (2.9%), and myocardial infarction (2.5%). Major complications included death (0.2%), emergent CABG (0.1%), stroke (<0.01%), tamponade (0.3%), and vascular complications (0.6%). In a more recent 2024 registry [23], the overall rate of major complications was reported to be similar, at approximately 1.7%.

While complications are relatively low, it is important to note that, on the other hand, suboptimal revascularization (defined as persistent significant side branch occlusion, or final TIMI flow grade 1 or 2, or residual diameter stenosis > 30%) is associated with higher rates of cardiac death and myocardial infarction compared to optimal revascularization [24]. This highlights the need for thorough evaluation of the patients’ potential benefit, as most of them present with stable angina [25].

## 5. Evidence from Studies

Several observational studies have compared successful and failed CTO-PCI, consistently showing benefits in the successful CTO-PCI groups. These include reductions in mortality and myocardial infarction rates, relief from angina, and fewer recurrent revascularizations [26,27,28,29]. In a meta-analysis by Christakopoulos et al. [30] including 14,876 patients, successful CTO-PCI was associated with lower mortality, reduced residual angina, a lower risk of stroke, less need for subsequent coronary artery bypass grafting (CABG), and a decreased risk of MACE. Additionally, other observational studies have demonstrated improvements in LVEF [31,32] and a reduction in ischemia burden [33], as assessed by SPECT, following CTO recanalization.

Randomized trials (RCT) are limited in the field of CTO. To date, only 6 major RCTs have compared CTO-PCI with optimal medical therapy (OMT). This lack of data contributes to the low level of evidence (B) in the 2018 European Society of Cardiology/European Association of Cardiothoracic Surgery guidelines [34]: “Percutaneous recanalization of CTOs should be considered in patients with angina resistant to medical therapy or with a large area of documented ischemia in the territory of the occluded vessel” (IIa/B). More recently, the 2021 ACC/AHA/SCAI Guidelines for Coronary Artery Revascularization [35] assigned a Class 2b recommendation for CTO PCI in patients with suitable coronary anatomy who continue to experience refractory angina despite optimal medical therapy and adequate treatment of non-CTO lesions.

The primary and key secondary outcomes of these studies are summarized in Table 1 [36,37,38,39,40,41,42,43,44]. With the exception of the DECISION-CTO trial, the other studies demonstrated a reduction in symptoms and an improvement in quality of life. It is to be noted that 20% of the patients in the OMT group received CTO-PCI within 3 days, suggesting that the OMT group might have benefited from the angina relief effect associated with PCI. The lack of difference in MACE outcomes in DECISION-CTO may be attributed to the study’s insufficient power, likely due to challenges in patient recruitment and slower enrollment, which prevented the trial from reaching an adequate sample size.

In the three-year follow-up of the EuroCTO trial, MACE were more frequent in the OMT group, primarily due to target vessel revascularization [37]. None of the trials demonstrated a reduction in mortality, as they were not designed or sufficiently powered to do so. Moreover, longer follow-up periods are needed to evaluate this outcome. Indeed, extended follow-up studies are required to assess potential reductions in mortality. In the Korean registry by Park et al. [9], which included 1547 patients followed for ten years, cardiac death was significantly higher in the OMT group compared to the CTO-PCI group, but only between 3 and 10 years. All-cause mortality also showed a similar trend. Similarly, in the Canadian registry [45], which included 1624 patients followed for ten years, patients were categorized based on whether they underwent CTO revascularization (PCI or CABG) or received OMT. A reduction in all-cause death was observed in the CTO-revascularization group. Another observational study from our group with an eight-year follow-up and comparing successful vs. failed CTO-PCI is ongoing [46]. However, in all these observational studies, the decision to perform or not perform CTO-PCI was based on the physician’s and patient’s choice, resulting in biases of patient selection.

Based on the current evidence, CTO revascularization should be considered for selected symptomatic patients, particularly those with angina or dyspnea and evidence of viable myocardium, to improve quality of life. However, the decision should be made collaboratively within the Heart Team, taking into account the patient’s age, comorbidities, presence of other coronary or valvular lesions, and, importantly, the patient’s preferences.

Non-invasive imaging plays a valuable role in selecting appropriate candidates for CTO PCI. Cardiac MRI and PET are particularly useful for assessing myocardial viability and scar burden, while nuclear stress testing and stress echocardiography help evaluate ischemia burden. Cardiac MRI uses late gadolinium enhancement to identify viable versus scarred myocardium and accurately quantify scar extent, which helps predict recovery potential after revascularization. PET imaging assesses both myocardial metabolism and perfusion, enabling detection of viable but underperfused myocardium (hibernating tissue) and ischemic areas that may benefit from intervention. Together, these imaging modalities support the Heart Team in making informed decisions on patient selection by identifying those most likely to benefit from CTO PCI.

## 6. Perspectives

The field of CTO has seen remarkable progress in recent years, supported by advancements in techniques and devices and peer proctoring. Yet, despite notable improvements in procedural success and symptom relief, the overall clinical benefit of CTO-PCI remains debated due to limited high-quality randomized data. Future progress will require a balanced integration of continued technical innovation and the generation of more robust clinical evidence.

Technological innovations continue to push the boundaries of what is feasible in CTO PCI. The refinement of microcatheters, re-entry devices like the Stingray system, and next-generation guidewires has significantly improved outcomes in complex lesions [47]. Advanced imaging techniques, particularly IVUS and coronary computed tomography angiography, play an increasing role in procedural planning, proximal cap ambiguity resolution, and post-intervention optimization. Looking ahead, the fusion of CCTA with real-time coronary angiography holds promise for improving visualization of the complex CTO anatomy and facilitating guidewire navigation through the occluded segment [48]. Clinically, the benefits of successful CTO PCI are well-documented in observational studies, which consistently show improvements in angina relief, quality of life, left ventricular function, and, in some cases, long-term survival. However, randomized controlled trials (RCTs) remain limited in number, size, and duration. Trials such as DECISION-CTO [40] and EuroCTO [41] have shown symptomatic benefit but failed to demonstrate reductions in major adverse cardiac events (MACE) or mortality, in part due to underpowering, crossover between treatment arms, and short follow-up periods. Furthermore, many trials have been challenged by slow enrollment, reflecting the complexities of patient selection and clinical equipoise in stable coronary artery disease. It is important to further consider that certain subgroups of patients, particularly those who are highly symptomatic, such as individuals with a proximal LAD CTO, may be less likely to be enrolled in randomized trials. These patients tend to experience significant clinical benefit from recanalization, as suggested by retrospective evidence. However, their relative exclusion from RCTs can lead to an underrepresentation of this population in randomized datasets. This underrepresentation may consequently dilute the observed treatment effect in RCTs, potentially underestimating the true benefit of intervention in these high-risk groups.

Additionally, the protective role of well-developed collateral circulation must be considered. In many patients, these collaterals provide sufficient perfusion to prevent myocardial infarction at rest, thereby reducing the likelihood that PCI will impact hard clinical endpoints within the stable population observed over short-to-intermediate follow-up periods. This physiological adaptation may further contribute to the diminished treatment effect seen in randomized trials, highlighting the complexity of interpreting outcomes across different study designs and patient populations.

Given these limitations, future RCTs must be adequately powered, methodologically rigorous, and include long-term follow-up, beyond 10 years, to truly assess the impact of CTO PCI on hard clinical outcomes, including mortality and myocardial infarction. These trials should also stratify patients by ischemia burden, viability, and symptom severity to better define the subgroups most likely to benefit.

## 7. Conclusions

CTO percutaneous coronary intervention has evolved into a safe and effective procedure when performed by experienced operators using modern techniques and dedicated equipment. While observational studies support improvements in symptoms, left ventricular function, and potentially long-term survival, current randomized evidence primarily demonstrates benefit in quality of life rather than hard clinical endpoints. Given the procedural complexity and associated risks, CTO-PCI should be reserved for carefully selected symptomatic patients with documented ischemia or viable myocardium. Decision-making should be multidisciplinary, patient-centered, and guided by a thorough assessment of anatomical feasibility, clinical benefit, and individual patient goals. Ongoing and future trials with extended follow-up are essential to clarify the long-term prognostic value of CTO revascularization.

## Figures and Tables

**Table 1:** Randomized controlled trials assessing patient outcomes with CTO-PCI compared to OMT.

Trials	Study Period	Population	Patients Included	Groups	Primary Endpoints Results	Secondary Endpoints Results
EXPLORE [36,37]	2007–2015	Patients with STEMI and concurrent CTO.	304	CTO-PCI vs. OMT	LVEF and LVEDV on cardiac MRI at 4 months: no difference between groups. In the ten-year outcomes EXPLORE trial, there was no difference in MACE (cardiac death, myocardial infarction, or CABG) at 10 years.	Infarct size and regional myocardial function: no difference between groups. Higher LVEF in LAD CTO-PCI group. No difference in MACE (cardiac death, myocardial infarction, or CABG) at 4 months. No difference in cardiac death at 4 months. In the ten-year outcomes EXPLORE trial, dyspnea relief was more frequent in the CTO-PCI group. No difference in all-cause death at 10 years.
REVASC [38]	2007–2015	Patients with symptoms and/or proof of ischemia.	205	CTO-PCI vs. OMT	Change in segmental wall thickening in the CTO territory at 6 months: no difference between groups.	Improvement of regional wall motion and changes in LV volumes and ejection fraction: no difference between groups. No difference in all-cause death at 12 months. Lower MACE (all-cause death, myocardial infarction, or any clinically driven repeat revascularization) at 12 months in the CTO-PCI group.
IMPACTOR-CTO [39]	2010–2014	Patients with stable angina and isolated RCA CTO.	94	CTO-PCI + OMT vs. OMT	Change in myocardial ischemia burden assessed with adenosine stress cardiac MRI at 12 months: lower in the CTO-PCI group.	Change in 6-min walk distance and QoL: improvement in the CTO-PCI group only. No difference in MACE (all-cause death, myocardial infarction, or unplanned revascularization) at 12 months.
DECISION-CTO [40]	2010–2016	Patients presenting with stable angina, silent ischemia, or ACS.	834	CTO-PCI + OMT vs. OMT	MACE (death, myocardial infarction, stroke, or any revascularization) at 4 years: no difference between groups.	No difference in QoL improvement in both groups (EQ-5D, SAQ). No difference in all-cause death at 4 years.
EURO-CTO [41,42]	2012–2015	Patients with symptoms and proof of myocardial viability.	396	CTO-PCI + OMT vs. OMT	Change in health status subscales as assessed by SAQ at 12 months: higher improvements in QoL, angina frequency, and freedom from angina subscales in the CTO-PCI group. In the three-year outcomes of the EURO-CTO trial, MACE was significantly higher in the OMT group (largely due to ischemia-driven revascularizations).	Better EQ-5D improvement in the CTO-PCI group Better CCS classification improvement in the CTO-PCI group. No difference in MACE (cardiac death, non-fatal myocardial infarction, and ischemia-driven target lesion revascularization) at 12 months. No difference in cardiac death at 3 years
COMET-CTO [43,44]	2015–2017	Patients with stable angina and/or proof of myocardial ischemia and/or proof of myocardial viability.	100	CTO-PCI + OMT vs. OMT	Change in QoL assessed with SAQ at 6 months: better improvements in all QoL subscales in the CTO-PCI group. In long-term, follow-up COMET-CTO trial, there was no difference in MACE (non-fatal myocardial infarction and recurrent revascularization) at 4.7 years.	No difference in LVEF improvement. No difference in MACE (non-fatal myocardial infarction and recurrent revascularization) at 275 days. No difference in cardiac death at 4.7 years.

ACS: acute coronary syndrome; CCS: Canadian Cardiology Society; LAD: left anterior descending; LVEF: left ventricular ejection fraction; LVEDV: left ventricular end diastolic volume; MACE: major cardiovascular events; MRI: magnetic resonance imaging; OMT: optimal medical therapy; PCI: percutaneous coronary intervention; RCA: right coronary artery; QoL: quality of life.

## Data Availability

No new data were created or analyzed in this study.
